# How education from children influences parents' green travel behavior? The mediating role of environmental protection commitment

**DOI:** 10.3389/fpsyg.2025.1532152

**Published:** 2025-04-29

**Authors:** Tong Zhang, Feiyu Chen, Xiao Gu, Zhuohang Li, Zhexin Zhu

**Affiliations:** School of Economics and Management, China University of Mining and Technology, Xuzhou, China

**Keywords:** green travel behavior, trans-education, environmental protection commitment, self-efficacy, reverse socialization

## Abstract

The transmission of green ideas within families is no longer limited to the older generations instilling ideas into the younger generations. Younger generations are increasingly influencing the green travel behavior of older generations with their green ideas. This study was based on social learning theory and reverse socialization theory and proposed the concept of “trans-education.” A theoretical model of green travel factors was constructed based on self-efficacy and environmental protection commitment (EPC) and its dimensions. By conducting a field experiment (*N* = 229) and administering a questionnaire survey across 31 provinces in China (*N* = 639), it has been found that: (1) Trans-education significantly contributes to residents' green travel behavior. This finding means transmitting green concepts from the younger to the older generation can positively influence green travel behavior. (2) Trans-education influences residents' green travel behavior through EPC and its dimensions. (3) Self-efficacy reduces the extent to which trans-education affects residents' green travel behavior. These findings offer new perspectives on the relationship between intergenerational transmission of green concepts within families and green travel behavior changes.

## 1 Introduction

Increasing energy consumption and greenhouse gas emissions present significant challenges to the global environment and the future sustainable development of humanity (Voosen, 2023). Therefore, it's crucial to guide a shift toward green travel in the general population (Jing et al., 2022). Green travel is an environmentally friendly and low-carbon behavior (Kumar et al., 2019), significantly reducing the energy consumption and pollution caused by urban transportation (Jia, 2018). To effectively encourage sustainable travel behavior and promote green transportation, it's essential to understand the underlying mechanisms. Such understanding is of great academic and practical importance for reducing carbon emissions, conserving energy resources, and promoting sustainable development.

Research has explored various strategies, such as government policies (Tu et al., 2023), infrastructure improvements (Liu and Tian, 2021), and technological innovation (Li et al., 2018), to encourage shifts in green travel behavior. These studies are rooted in a comprehensive understanding of the psychological drivers of green travel behavior. Norm activation theory and the theory of planned behavior provide key insights into the factors influencing individuals' choices (Lanzini and Khan, 2017; Yang et al., 2020). However, the reality is that while many individuals have the intention to travel green, they often fail to put it into practice (Geng et al., 2017). This suggests that additional methods and approaches are necessary to promote green travel behavior transformation effectively.

When discussing motivations behind green travel behavior, it is important to note that family structure is often considered a significant control variable (Chen and Liang, 2016). However, while Yang et al. (2017) have found differences in green travel choices among families with different structures, they have not yet delved into the underlying mechanisms. Previous studies in the field of green and low-carbon behavior have acknowledged the transmission effect between generations within a family (Wang et al., 2022). However, whether children can influence their parents' green practices remains contested as variations in research methodologies have led to inconsistent findings among scholars (Jaime et al., 2023; Wang and Li, 2024). As intergenerational family interactions become more frequent (Deng et al., 2022), the younger generation plays a crucial role in shaping green and low-carbon behavior. The reverse socialization theory provides a basis for the younger generation influencing their parents' green travel behavior. The reverse socialization theory mentions that in times of rapid change, the older generation will engage in extensive cultural absorption from the younger generation (Zhou, 2000). Chen et al. (2025) proposed the concept of trans-education, defining it as the process by which children provide environmental education to their parents. This study adopts this concept to explain the process through which children transmit green concepts to their parents within the family. Given the critical role of observational learning in behavioral change, as emphasized by social learning theory (Gao, 2000), this study argues that trans-education can profoundly influence the green travel behavior. This study uses field experiments to confirm that trans-education can affect residents' green travel behavior. Afterwards, a questionnaire survey was conducted to expand the experimental subjects nationwide for verification, clarifying the impact mechanism of trans education on residents' green travel behavior.

Individuals often practice or give up green and low-carbon behavior for various reasons (Vlasceanu et al., 2024). How do we reflect on whether the influence of trans-education on green travel behavior is long-term and sustainable? Commitment, an important concept in psychology, represents the determination a person has toward achieving a specific goal or behavior. This study applies the commitment variable to green travel behavior to reflect on a resident's persistent willingness to protect the environment and clarify the trans-education mechanism on green travel behavior. Researchers have divided commitment dimensions when using it as a variable (Lu et al., 2023). The emotional contagion and knowledge transmission have been confirmed by scholars as the pathways through which trans-education influences green and low-carbon behavior (Wang and Li, 2024; Singh et al., 2020). However, limited research has explored the role of normative commitment and its potential influence in this process. Building on psychological factors such as environmental attitudes (Cauwelier et al., 2024), environmental perceptions (Sabbir and Taufique, 2022), and ethical norms (Wang et al., 2023) (proved by scholars to be predictive of green travel behavior), this study introduces the concept of the environmental protection commitment (EPC) to better understand and apply the commitment variable to environmental behavior. EPC reflects an individual's commitment to green behavior and environmental goals, and represents an individual's loyalty to environmental protection actions. The EPC will measure a human's loyalty to the environment and reflect individual loyalty to environmental protection and the determination to maintain long-term environmental protection behavior. Moreover, EPC can be classified into three categories: emotional commitment (EC), normative commitment (NC), and continuous commitment (CC), to clarify the role of trans-education.

As pointed out by social learning theory, behavioral change is not only influenced by observational learning (Gao, 2000) but by self-initiated behavior results, i.e., self-efficacy. As seen in studies on green behavior, the strength of the public's belief and confidence in their green behavior can impact their behavioral choices (Lin and Hsu, 2015). After undergoing trans-education, self-efficacy may moderate the effect of trans-education on green travel behavior. For individuals with lower levels of self-efficacy, they are often less likely to easily alter their current travel habits (Shehawy, 2023). This research uses social learning theory to explore the role of self-efficacy in the influence mechanism of trans-education on green travel behavior.

Based on reverse socialization and social learning theory, this study used empirical analyses to explore how trans-education affects residents' green travel choices and investigate self-efficacy's role. The introduction of variable EPC helps to predict and motivate the public's environmental behavior. By further exploring the various dimensions of environmental commitment variables, we provide in-depth insights for the comprehensive development and sustainable choice of green travel. This study aims to fill in the gaps in existing research, provide targeted suggestions for policymakers and practitioners, and guide residents to choose green modes of transport more effectively. This promotes sustainable development.

## 2 Literature review

### 2.1 Trans-education and its relationship with green travel behavior

Communication within the family is two-way; the process by which the older generation follows the younger generation in knowledge acquisition is called “post-metaphorical culture.” Zhou (2000) referred to “post-metaphorical culture” as “reverse socialization,” and used the term “cultural feedback” to describe the following phenomenon: “In the era of rapid social changes, the older generation has extensively absorbed culture from the younger generation” (Zhou, 2011). Based on the above study, it is easy to speculate that children's green perceptions may influence their parents' green travel behavior in families.

Much research has been carried out on green and low-carbon behavior, focusing on the impact that other people's green behavior has on an individual. Cialdini et al. (1990) found that other people's non-green behavior would lead to other individual's non-green behavior. Schultz et al. (2007) noticed that a stranger's green and low-carbon behavior would impact others. A more in-depth study by Meijers et al. (2019) confirms the positive effect that the green consumption behavior of friends or colleagues has on an individual's behavior. Griskevicius et al. (2012) showed that individuals can influence their behavior by observing the green and low-carbon behavior of others. It can be found that all of these studies explore the influence of members outside the household on the behavior of the residents themselves. Cakanlar et al. (2023) found there is an influence mechanism of green and low-carbon behavior between partners. At the same time, he also pointed out that few studies have explored the influence of green and low-carbon behavior of internal family members on individual behavior choices. Returning to the field of green travel research, researchers have used TPB, SCT, and other theories to predict many influencing factors when exploring green travel. In these studies, family structure or family members are often considered control variables. When discussing the influence of family heterogeneity on green travel behavior, Luo et al. (2022) pointed out that family size significantly impacts individual green travel behavior. Echeverría et al. (2022) found that different family situations could affect the green travel behavior of an individual. Furthermore, Yang et al. (2017) found a significant correlation between whether there were children in the family and whether residents had green travel behavior. This suggests that there may be intergenerational influences from children, within the family, on the green travel behavior of residents. Although previous studies have explored these topics from various perspectives, they have provided only limited insight into the mechanisms of mutual influence among family members, lacking a comprehensive analysis of intergenerational influence dynamics. Therefore, this study starts from an intergenerational perspective and explores how children's green perceptions affects their parent's choices.

Wang et al. (2022) pointed out that there is a transmission effect of green and low-carbon behavior between generations within the family. Singh et al. (2020) found that when children are more concerned about green and low-carbon behavior, their parents will have better environmental protection attitudes. However, children's objective knowledge of the environment does not influence their parents' green and low-carbon behavior. Jaime et al. (2023) argued that children's environmental education is not effective in improving their parents' green behavior. This suggests that children may not have an impact on their parents green and low-carbon behavior. The above differences may be attributed to different methods used by scholars for exploration. Therefore, this study adopts multiple methods to test the mechanism of trans-education effects on residents' green travel behavior. Today there is growing evidence that children's environmental education of their parents is effective in improving their parents' green and low-carbon behavior. Ding et al. (2024) verified that parent-child interactions are effective in facilitating the emergence of green and low-carbon behavior in families. In summary, scholars have explored the factors within the household that may influence residents' green travel behavior, but there is a lack of research on whether and how children can influence their parents' green travel behavior. According to social learning theory (Gao, 2000), observational learning positively affects behavioral change. When the older generation follows the younger generation in adopting green concepts, they may show a tendency toward green travel behavior. This study proposes the following hypothesis.

H1: Trans-education has a significant impact on green travel behavior.

### 2.2 The intermediary role of the environmental protection commitment

In a related study on commitment as a variable, Ling et al. (2001) defined organizational commitment as an employee's attitude toward the organization and identified it as an important indicator of loyalty to a company. In this study, the commitment variable is expanded to the field of environmental behavior, and the concept of EPC is put forward. As a key indicator to test environmental loyalty, EPC reflects the individual's commitment to green behavior and environmental protection goals. This shows the positive psychological characteristics of individuals participating in environmental protection activities.

As Ling et al. (2012) pointed out, the commitment variable contains multiple dimensions. Our predecessors analyzed the psychological factors affecting green travel from multiple angles. Shi (2019) found that public environmental awareness can affect green travel behavior, while Khan (2023) focused on the positive impact of environmental attitudes on green travel behavior. Ru et al. (2018) confirmed the positive effect of ethical norms on individual green travel behavior intentions, and Si et al. (2020) affirmed the importance of moral norms. However, no research builds a unified psychological framework that summarizes the psychological variables of residents. The EPC variable is easy to divide into dimensions, helping to unify various influencing factors. Since the EPC comes from three areas: the individual's environmental emotion, interest exchange, and moral norms, the EPC is defined in three dimensions: the emotional commitment (EC), continuous commitment (CC), and normative commitment (NC) dimensions. EC refers to the public's emotional dependence on a good environment, their recognition of the value and goals of environmental protection behavior, and their willingness to achieve environmental goals. CC refers to the public's awareness of the potential losses caused by environmental degradation and the requirement of long-term action to protect the environment. NC reflects the public's obligation to protect the environment, that is, the social responsibility formed due to long-term social impacts and the public's commitment to protecting the environment. Overall, the level of EPC will interfere with individual behavioral choices. Based on the above analysis, this paper proposes the following hypotheses.

H2: EPC plays an intermediary role in the process of trans-education affecting green travel behavior.

H2a: Trans-education has a significant positive effect on EPC.

H2a-1: Trans-education has a significant positive effect on EC.

H2a-2: Trans-education has a significant positive effect on NC.

H2a-3: Trans-education has a significant positive effect on CC.

H2b: EPC has a significant positive effect on green travel behavior.

H2b-1: EC has a significant positive effect on green travel behavior.

H2b-2: NC has a significant positive effect on green travel behavior.

H2b-3: CC has a significant positive effect on green travel behavior.

### 2.3 The moderating effect of self-efficacy

Green travel behavior typically requires changes in daily transportation choices and travel patterns, requiring a shift in personal travel modes—a change that is often difficult to implement. Self-efficacy may influence the effectiveness of trans-education in promoting green travel behavior among residents. Self-efficacy refers to an individual's self-assessment of their ability to perform a particular behavior and achieve the desired outcome (Gao, 2000). Shehawy (2023) found that individuals with low self-efficacy, despite having a strong intention to engage in green behavior, may fail to exhibit such behavior. Similarly, Qin et al. (2024) observed that green self-efficacy moderates the relationship between future self-continuity and pro-environmental behavior in middle school students. However, this moderating effect was not significant among high school students. Trans-education transmits green knowledge from children to parents and may influence residents' green travel behavior, with self-efficacy acting as a moderating factor in this process. It can be inferred that, following the intergenerational transmission of green concepts, individuals' levels of self-efficacy will intervene in their choice of green travel behavior. Therefore, this study proposes the following hypothesis:

H3: The direct predictive effect of trans-education on green travel behavior is moderated by self-efficacy.

**Figure 1:**
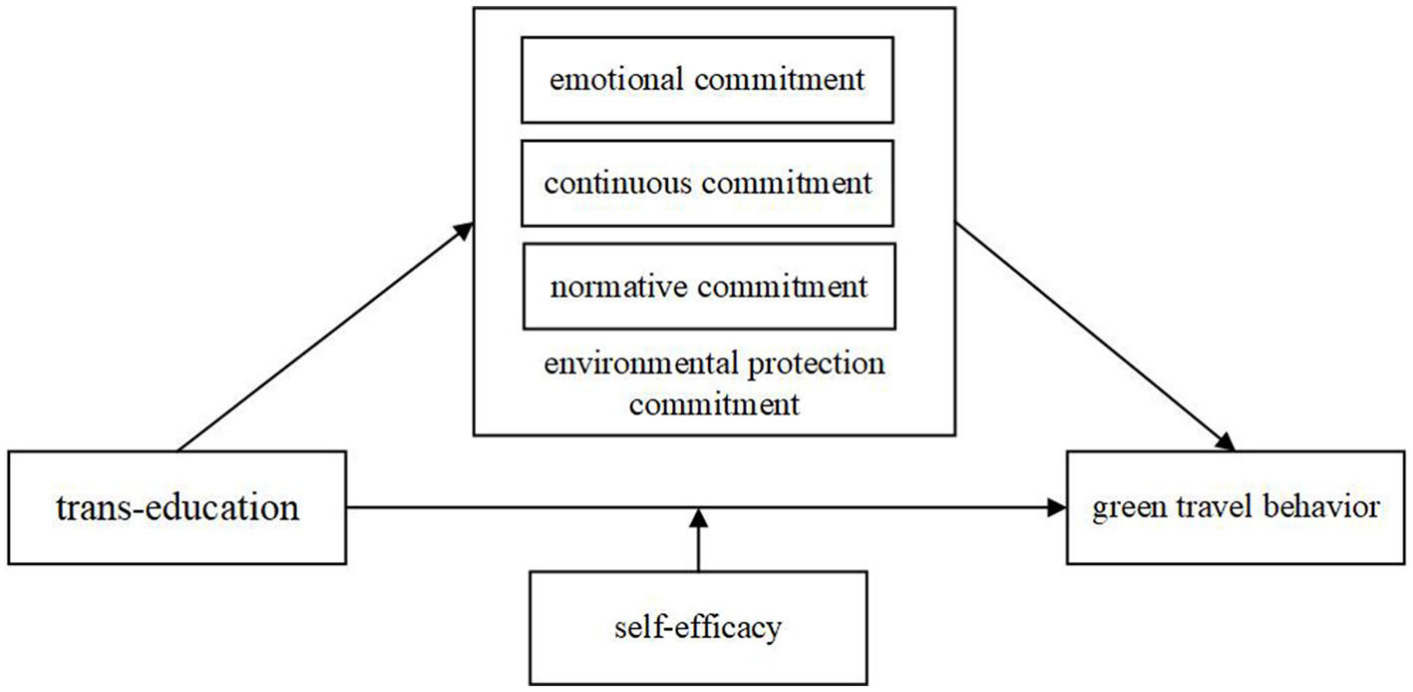
Concept model.

In summary, this study proposes the theoretical model seen in Figure 1.

## 3 Methods

### 3.1 Study I

To confirm the causal relationship between trans-education and residents' green travel behavior, we invited 251 parent-child pairs to participate in a carefully designed field experiment. This experiment was aimed to prove our argument that there is a strong relationship between trans-education measures and the promotion of residents' green traveling behavior. This experiment aims to demonstrate our argument that there is a close relationship between cross educational measures and promoting residents' environmentally friendly travel behavior.

#### 3.1.1 Experimental design

We determined one primary schools in Jinan, Shandong province as the subjects of our study. Jinan, located in northern China, is the capital city of Shandong Province and boasts a relatively advanced economy and high level of urbanization. As a regional transportation hub, Jinan has well-developed travel infrastructure, including an extensive public transportation system such as buses and subways, offering residents a diverse range of commuting options. Upon enrolment, these children were randomly divided into different classes, ensuring variation in the personal characteristics of the students' parents (Otto et al., 2019). There was a positive correlation between the grade of the children and the age of their parents, with a total of 229 parents in the experiment (N _controlgroup_ = 102, N _experimentgroup_ = 127), all in grades one to six of which 101 (44.1%) were fathers of the children and 128 (55.9%) were mothers of the children.

We have designed two sets of questionnaires, respectively known as the pre-questionnaire and the post-questionnaire. The pre-questionnaire is primarily utilized to document the demographic characteristics of parents and their green travel behavior prior to the intervention. Our study used the frequency of recording residents' six green travel mode choices in the past week to measure residents' green travel behavior (Echeverria et al., 2022). The post-questionnaire is administered to parents after conducting green and low-carbon education for children in the experimental group. It aims to record the impact of the trans-education and their green travel behavior afterward. The trans-education scale borrowed from the scale developed by Singh et al. (2020) to measure how young people environmentally reverse socialize their parents.

By explaining the guidelines for green and low-carbon living, we informed the children of what green travel behavior entail and how they can be implemented. We had positive interactions and exchanges with the children, and ultimately demonstrated to them how to adopt more green and low-carbon practices in their travel chose. One week after educating the children about green and low-carbon practice, a post-questionnaire was sent to the parents. Children in the control group were not educated about green and low carbon and the post-questionnaire given to their parents was only used to investigate green and low carbon behavior.

#### 3.1.2 Experimental results

**Figure 2:**
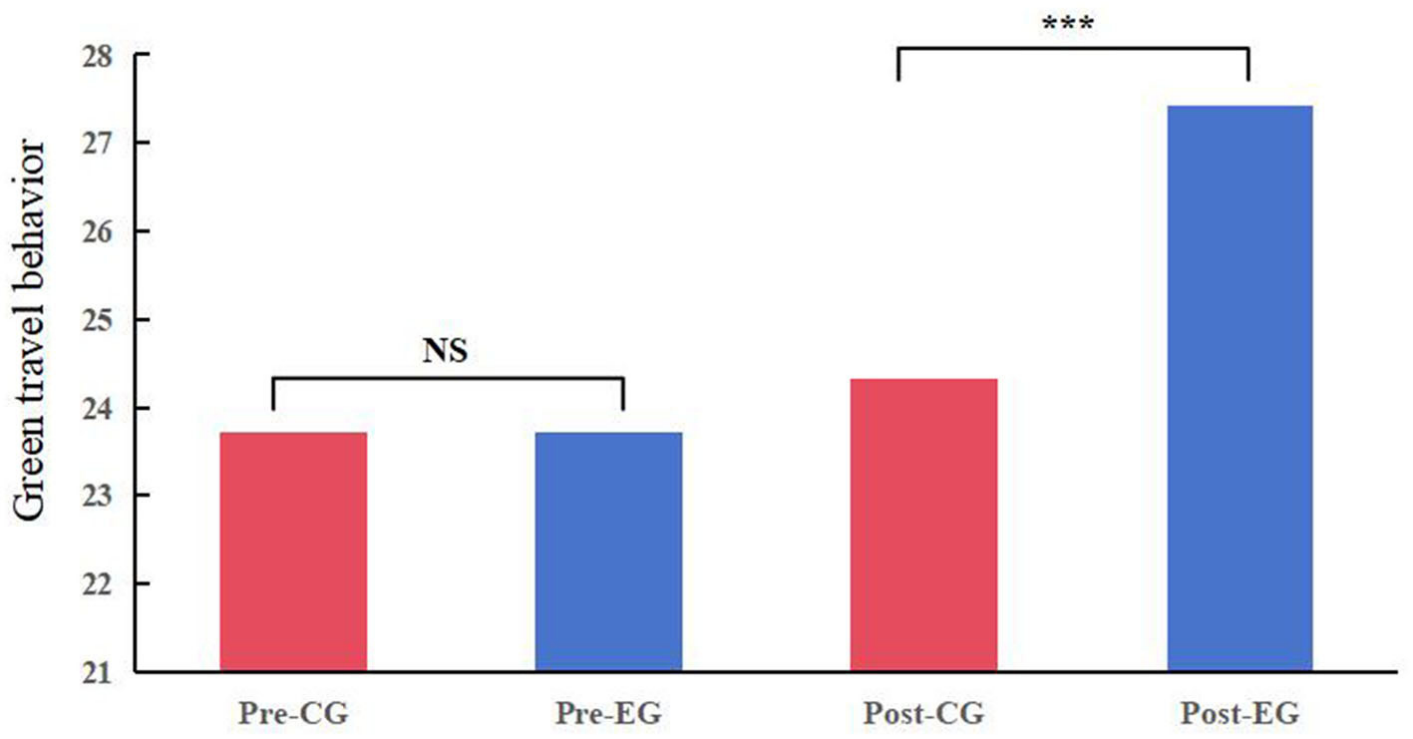
Levels of green travel behavior in the experimental and control groups at different time points. Pre-CG, control group before intervention; Pre-EG, experimental group before intervention; Post-CG, control group after intervention; Post-EG, experimental group after intervention; NS, no Significance; ***, significant at the 1% level.

The results of Study I are presented in Figure 2, the parents in the experimental and control groups exhibited comparable green travel performances in the past week, indicating a similar baseline prior to intervention (M _control group_ = 23.724, M _experiment group_ = 23.726, *p* = 0.999). A total of 195 (85.15%) parental post-questionnaires were collected from students in the experimental group and control group. The average score of trans education in the experimental group was 18.790 (significantly exceeding the critical value of 10 points, SD = 1.850, T = 46.302, *P* < 0.001). This indicates that the experimental group students have successfully conveyed the concept of green and low-carbon to their parents. Post-education analysis revealed a noteworthy upsurge in green travel behavior among parents of students in the experimental group, as compared to the control group. Specifically, the experimental group's mean score escalated to 27.414, outperforming the control group's 24.320 (t = 6.868, *P* < 0.001). This transformation hints at the occurrence of trans-education, a phenomenon where children' green concept ripple out to influence their parents' green travel behavior. We delved into the correlation between trans-education and the green travel in the experimental group. Our findings unequivocally indicate that trans-education has a significant impact on fostering green and low-carbon behavior among parents (R^2^ = 0.538, F = 108.461, B = 0.734, *P* < 0.001). Thus, we strengthened our validation of H1, confirming the potential of students to act as catalysts for green travel behavior within their families.

### 3.2 Study II

#### 3.2.1 Variable measurement

The hypothesis of the relationship between trans-education, self-efficacy, EPC, and green travel behavior has been proposed in the previous section. Scale design and development are needed to measure each variable better and verify the hypotheses. This study's trans-education Scale consistent with Study I. The self-efficacy measurement scale was borrowed from a well-established scale, revised the relevant questions in the questionnaire to consider the current research context, as well as considering the textual expressions in the Chinese background (Bian, 2004). For example, the question items include: “I believe that my personal environmental protection behavior help the earth become a better place” and “I believe that I can change my non-environmental protection behavior, so as to better protect the environment.” The EPC scale is based on the OCR scale in the field of organizational management, which is expanded and revised to the field of environmental behavior (Ling et al., 2001).

The current literature outlines three methods for measuring green travel behavior: travel frequency, time, and distance. The method of sorting measurements in this study measures travel frequency (Geng et al., 2016). For the five modes of transport (walking, public transportation, shared bicycle, private car, taxi/rental) used by people for daily travel, according to the daily uses for the respondents to rank, the comprehensive score of the mode is (6-ranking) ^*^ (±1) points, in which the green mode of travel is +1 points. In contrast, the non-green mode of travel is −1 points. The arithmetic shows that the green travel score = 6 – walking – public transportation trips – shared bicycle trips + private car trips + taxi/rental trips (ranked). The score has seven cases: the score is “−3” when the non-green travel behavior is in first and second place; “−1” when the non-green travel behavior is in first and third place; “1” when the non-green travel behavior is in first and fourth place; “3” when the non-green travel behavior is in first and fifth place; “1” when the non-green travel behavior is in second and third place; and 1 when the non-green travel behavior is in second and fourth place. Non-green trips in second and third place are scored 1-point, non-green trips in second and fourth place are scored 3 points, non-green trips in second and fifth place are scored 5 points, non-green trips in third and fourth place are scored 5 points, non-green trips in third and fifth place are scored 7 points, non-green trips in fourth and fifth place are scored 9 points. As shown in Table 1.

**Table 1:** Examples of scores.

**Score**	**Situations**
−3	Non-green travel modes ranked as first and second, respectively
−1	Non-green travel modes ranked as first and third, respectively
1	Non-green travel mode rankings as first and fourth, or second and third, respectively
3	Non-green travel mode rankings as first and fifth, or second and fourth, respectively
5	Non-green travel modes ranked as third and fourth, respectively
7	Non-green travel modes ranked as third and fifth, respectively
9	Non-green travel modes ranked as fourth and fifth, respectively

#### 3.2.2 Data sources

Study II used Credamo, an online data collection platform, to conduct data collection in China. In order to ensure the reasonableness of the study, the target population of this study consists of individuals with children and households owning private vehicles that are fueled by gasoline. Therefore, when distributing the questionnaire, the screening criteria were set as follows: “Do you have children?” and “Is your private vehicle a gasoline-powered car?” A total of 702 questionnaires were distributed in this study, and 639 valid questionnaires were recovered (after excluding invalid questionnaires such as “selecting all the same options”), with a valid recovery rate of 91.17%.

## 4 Results

### 4.1 Reliability and validity analysis

The reliability and validity of the formal questionnaire were tested using SPSS version 27.0, and the results are shown in Table 2. The Cronbach's alpha coefficient of trans-education, EC, NC, CC, EPC, and self-efficacy are all >0.6, and the scale is reliable. This study strictly follows the procedure of scale development. We repeatedly discussed this with two management experts and made the scale corrections based on literature studies. Therefore, the scale has good content validity, and the main form of test in terms of construct validity is the KMO value. The results of Bartlett's spherical test are shown in Table 2. The KMO of each variable is >0.6, the chi-square of Bartlett's spheres is larger, and the results are statistically significant (Sig < 0.01), indicating that this scale is suitable for factor analysis.

**Table 2:** Results of the confidence analysis.

**Variable**	**Cronbach's α**	**Number of topics**	**KMO**	**Sig**
Trans-education	0.803	4	0.796	<0.01
EC	0.732	3	0.664	<0.01
NC	0.668	3	0.663	<0.01
CC	0.672	3	0.644	<0.01
EPC	0.891	9	0.874	0.000
Self-efficacy	0.789	4	0.768	<0.01

### 4.2 Correlation analysis

The Pearson relationship between the resident's EPC, EC, CC, NC, self-efficacy, and trans-education is shown in Table 3, showing that resident's trans-education is positively correlated with their EPC, EC, CC, and NC at the 0.01% significance level, i.e., the more trans-education a resident has received, the more likely they are to be emotionally loyal and have a proactive, sustained and normative loyalty to environmental protection behavior. Hypotheses H2a, H2a-1, H2a-2, and H2a-3 were preliminarily verified as correct. The Eta squared coefficient between residents' trans-education, EC, CC, NC, self-efficacy, and EPC, and their green travel behavior is shown in Table 4; this indicates that green travel behavior is strongly correlated with trans-education, self-efficacy, EPC, EC, NC, and CC at the 0.01% significance level. Hypotheses H1, H2b, H2b-1, H2b-2, and H2b-3 were preliminarily verified as correct.

**Table 3:** Correlations between variables.

**Pearson relationship**	**EPC**	**EC**	**CC**	**NC**		**Self-efficacy**
Trans-education	0.822^**^	0.786^**^	0.761^**^	0.720^**^		0.823^**^
Eta^2^	Trans-education	EC	CC	NC	EPC	Self-efficacy
Green travel behavior	0.284^**^	0.223^**^	0.265^**^	0.265^**^	0.343^**^	0.200^**^

*** *P* < 0.005,

** *P* < 0.01,

* *P* < 0.05.

**Table 4:** The mediating role of environmental protection commitment on the path of influence of trans-education on green travel behavior.

**Variables**	**Green travel behavior**				**EPC**	
	**Model 1**		**Model 2**		**Model 3**	
	**B**	**t**	**B**	**t**	**B**	**t**
Trans-education	0.537	12.416^***^			1.618	36.378^***^
EPC			0.267	12.102^***^		
R^2^	0.195		0.187		0.675	
F	154.169^***^		146.459^***^		1,323.360^***^	

*** *P* < 0.005,

** *P* < 0.01,

* *P* < 0.05.

### 4.3 Regression analysis

#### 4.3.1 Analysis of the mediating effect of EPC and its dimensions

As the hierarchical variable category is greater than five, linear regression can be used for the analysis (Liu et al., 2013). To verify the mediating effect of EPC and its sub-dimensions in trans-education's influence on green travel behavior, this study applies hierarchical regression and verifies them separately.

##### 4.3.1.1 Analysis of the mediating effect of EPC

Using the input method, for the regression analysis of the influence effect between trans-education and green travel behavior, the regression model is recorded as model 1. For the regression analysis of the influence effect between EPC and green travel behavior, the regression model is recorded as model 2. For the regression analysis of the influence effect between trans-education and EPC, the regression model result is recorded as model 3. The results are shown in Table 4. The regression model demonstrates a significant mediating effect of EPC with a high R^2^ value (R^2^ = 0.675, *P* < 0.005), confirming hypotheses H1, H2a, and H2b.

##### 4.3.1.2 Analysis of the mediating effect of EC, NC and CC

Using the input method, for the regression analysis of the influence effect between trans-education and green travel behavior, the regression model is recorded as model 1. For the regression analysis of the influence effect between EC and green travel behavior, the regression model is recorded as model 2. For the regression analysis of the influence effect between trans-education and EC, the regression model result is recorded as model 3. The results are shown in Table 5. The regression model demonstrates a significant mediating effect of EC with a high R^2^ value (R^2^ = 0.617, *P* < 0.005), so hypotheses H2a-1 and H2b-1 are valid.

**Table 5:** The mediating role of emotional commitment on the path of influence of trans-education on green travel behavior.

**Variables**	**Green travel behavior**				**EC**	
	**Model 1**		**Model 2**		**Model 3**	
	**B**	**t**	**B**	**t**	**B**	**t**
Trans-education	0.537	12.416^***^			0.543	32.036^***^
EC			0.624	9.564^***^		
R^2^	0.195		0.126		0.617	
F	154.169^***^		91.470^***^		1,026.309^***^	

*** *P* < 0.005,

** *P* < 0.01,

* *P* < 0.05.

Using the same method to analyze the mediating effect of NC, the regression model result is recorded as model 3. The results are shown in Table 6. The regression model demonstrates a significant mediating effect of NC with a high R^2^ value (R^2^ = 0.519, *P* < 0.005), so hypotheses H2a-2 and H2b-2 are valid.

**Table 6:** The mediating role of normative commitment on the path of influence of trans-education on green travel behavior.

**Variables**	**Green travel behavior**				**NC**	
	**Model 1**		**Model 2**		**Model 3**	
	**B**	**t**	**B**	**t**	**B**	**t**
Trans-education	0.537	12.416^***^			0.517	26.195^***^
NC			0.663	10.728^***^		
R^2^	0.195		0.153		0.519	
F	154.169^***^		115.100^***^		686.173^***^	

*** *P* < 0.005,

** *P* < 0.01,

* *P* < 0.05.

For the analysis of the mediating effect of CC, the same method was applied. The results are shown in Table 7. The regression model demonstrates a significant mediating effect of CC with a high R^2^ value (R^2^ = 0.617, *P* < 0.005), so hypotheses H2a-3 and H2b-3 are valid.

**Table 7:** The mediating role of continuous commitment on the path of influence of trans-education on green travel behavior.

**Variables**	**Green travel behavior**				**CC**	
	**Model 1**		**Model 2**		**Model 3**	
	**B**	**t**	**B**	**t**	**B**	**t**
Trans-education	0.537	12.416^***^			0.558	29.603^***^
CC			0.737	12.508^***^		
R^2^	0.195		0.197		0.579	
F	154.169^***^		156.446^***^		876.347^***^	

*** *P* < 0.005,

** *P* < 0.01,

* *P* < 0.05.

##### 4.3.1.3 Analysis of the moderating effect of self-efficacy

Before analyzing the moderating effect, the degree of multicollinearity between the variables is investigated. This study examined the Pearson correlation coefficients between the moderating and other variables. The results showed that the coefficients were all <0.75. The VIF coefficients were all strictly <5, meaning the multicollinearity is not serious, and the moderating effect analysis can be carried out. Using this method of testing regression coefficients, we tested the moderating effect of self-efficacy in trans-education on green travel behavior (Wen et al., 2004). Three models were constructed separately using trans-education as the independent variable, green travel behavior as the dependent variable, and self-efficacy as the moderating variable while calculating the interaction term. The results are shown in Table 8. The significance of the amount of change in R^2^ is 0.006 < 0.05, and the significance of the interaction term is 0.04 < 0.05. The results indicate that self-efficacy significantly moderates the impact of trans-education on green travel behavior. Therefore, hypothesis H3 is valid.

**Table 8:** Analysis of the moderating effects of self-efficacy.

	**Model 1**			**Model 2**			**Model 3**		
**Variables**	**B**	**Standard error**	**t**	**B**	**Standard error**	**t**	**B**	**Standard error**	**t**
Trans-education	0.537	0.043	12.416^***^	0.470	0.076	6.162^***^	0.787	0.170	4.638^***^
Self-efficacy				0.091	0.084	1.076	0.341	0.146	2.332^**^
Interaction term							−0.022	0.010	−2.092^*^

*** *P* < 0.005,

** *P* < 0.01,

* *P* < 0.05.

**Figure 3:**
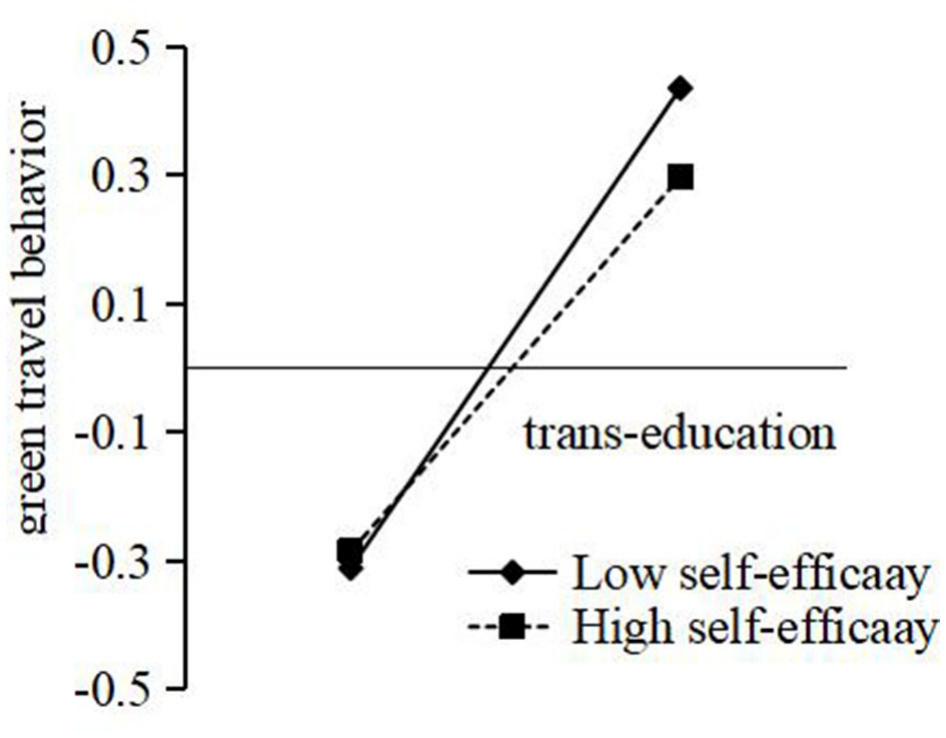
The moderating role of self-efficacy.

Another simple slope test was conducted on the above moderating effect to further study the trend of each variable under the moderating effect. Figure 3 shows the simple slope plot; compared with high self-efficacy, people's trans-education with low self-efficacy can better promote green travel behavior. The moderating effect is significant.

## 5 Discussion

### 5.1 Trans-education and green travel behavior

It was found that trans-education significantly influences green travel behavior, suggesting that the transmission of green ideas from the younger generation to the older generation impacts the older generation's choice of travel. This finding confirms the influence of another person's green behaviors on individual decision-making (Meijers et al., 2019). It explains why family heterogeneity affects green travel decisions (Yang et al., 2017). The younger generation receives more green information from the outside world than the older generation (Bulut et al., 2017), leading to stronger green perceptions. In intergenerational family communication, younger generations transmit more up-to-date green concepts to the older generation through trans-education (Zhou, 2000). As green concepts of the older generation become stronger, their travel behaviors shift toward more environmentally friendly transportation choices. This finding provides a new perspective for understanding the individual-level factors influencing green travel, complementing and improving the existing research framework. Our research findings differ from those of Jaime et al. (2023), possibly due to the fact that our study was conducted in different cultural environments. Consequently, it underscores the importance of exploring alternative research methodologies, such as laboratory experiments and informational intervention trials, to further validate our findings and ensure a more comprehensive understanding in the future.

### 5.2 The mediating role of EPC

Children's influence on their parents is more emotional than intellectual, such as ethics and norms (Wang et al., 2023; Chen et al., 2025). Trans-education is both a direct expression of children's ethical behaviors and a process of parental education. Therefore, it can influence residents' green travel behaviors by affecting a resident's EPC. Commitment often represents a longer and more active choice, consequently, trans-education makes residents more active in choosing a low-carbon and environmentally friendly way of traveling. After analyzing the role of different dimensions of EPC, it was found that EC, CC, and NC all played a mediating role. In the emotional dimension, EC serves as a mediator, as trans-education can strengthen individuals' emotional attachment to and identification with environmental protection behaviors. When children show greater concerned for green and low-carbon behaviors, their parents will develop more positive environmental protection attitudes (Singh et al., 2020). They recognize the value and goals of environmental protection behaviors, fostering voluntary environmental protection in such behaviors (Khan, 2023). NC serves as a mediator because trans-education can enhance residents' sense of responsibility for environmental protection behaviors. Parents among residents tend to be more prone to embracing green and low-carbon practices, a phenomenon often referred to as the “green parent effect” (Shrum et al., 2023). A profound sense of accountability toward future generations serves as a pivotal catalyst in fostering such environmentally conscious behaviors among residents (Syropoulos et al., 2023). This sense of responsibility forms a soft constraint on the residents' green travel choices, and they will pay attention to whether their behaviors are environmentally friendly (Wang et al., 2023). At the same time, they will consider whether it will affect the development of their children's environmental protection behaviors, which in turn motivates them to choose a green mode of transport. In the persistence dimension, CC serves as a mediator, as trans-education promotes a sense of benefit acquisition for residents' green travel behaviors. The older generation's environmental protection behaviors will influence the younger generation's environmental protection behaviors (Singh et al., 2020), and the process of trans-education shows that their education is successful. This psychological satisfaction makes the older generation more persistent and proactive in choosing green modes of transport (Wang et al., 2021). Given the longevity of commitment and the comprehensive nature of its dimension, trans-education has a more enduring impact on residents' choice of green transportation modes by enhancing their effectiveness, persistence, and normality.

### 5.3 The moderating effects of self-efficacy

Self-efficacy plays a moderating role in the path of influence of trans-education on green travel behavior. This verifies that the level of individual self-efficacy shows willingness (Lin and Hsu, 2015). However, unlike previous studies, this study found that excessively high self-efficacy can inhibit the effect of trans-education on green travel behavior and thereby hinder the adoption of green transportation modes. Self-determination theory and moral disengagement theory provide a robust explanation for this phenomenon. Self-determination theory posits that individual behavior is driven by both intrinsic and extrinsic motivation, whereas moral disengagement theory suggests that individuals may engage in unethical behavior to fulfill specific goals or needs, despite recognizing violations of moral standards. In this study, transgenerational education represents a process in which children educate their parents. For parents with high self-efficacy, the belief in their ability to independently engage in green and low-carbon behavior is stronger than merely adjusting their travel modes. Consequently, they are more likely to rationalize their non-green travel behaviors, further weakening the impact of transgenerational education on green travel behavior.

### 5.4 Limitations

This study has several limitations: (1) Given that communication between primary school students and their parents is more frequent and effective, this study selected parent-child pairs from a primary school in Jinan, Shandong. Additionally, the survey targeted households with private vehicles. Future research could expand the scope of field experiments by including families from diverse economic and cultural backgrounds to enhance the generalizability of the findings. (2) This study adopted travel frequency as a measure of residents' green travel behavior, following Geng et al. (2017) research. Future studies could develop a more comprehensive, multidimensional measurement framework to better capture variations in green travel behavior.

## 6 Conclusion

This study explored the effect of trans-education on green travel behavior using a linear regression model; the results showed a significant positive association between the two. When the value of trans-education increases, the expected value of green travel behavior increases accordingly. This effect is statistically significant. This reveals the positive impact of green perceptions transmitted by the younger generation to the older generation on their travel choices. As the younger generation's green perceptions increase, the older generation is influenced by them and will be more inclined to choose more environmentally friendly modes of travel.

To further explore the intrinsic mechanisms in this influence pathway, the study used hierarchical regression to test the mediating role of EPC and its three dimensions (EC, NC, and CC) between trans-education and green travel behavior, respectively. The study results indicate that trans-education significantly affects EPC and its dimensions, with the expected value of these commitments increasing as trans-education strengthens. EPC encapsulates residents' cumulative attitudes and steadfast commitments toward environmental actions, suggesting a heightened likelihood of consistent adoption of green travel behaviors. EC serves as a mirror, reflecting residents' emotional tendencies toward environmental behaviors and revealing their views on the environmental protection. Trans-education fosters more positive emotional attitudes toward green travel behaviors. From the NC perspective, trans-education fortifies the societal norms and constraints that guide individuals' environmental practices, thereby encouraging conformity to eco-friendly behaviors. Simultaneously, Trans-education enhances residents' CC, empowering them to recognize the intrinsic value and tangible benefits associated with selecting green travel modes. Therefore, EPC and its dimensions (EC, NC, and CC) partially mediate trans-education and green travel behavior.

In addition, self-efficacy was found to play a moderating role in trans-education and green travel behavior. Although both the change in R^2^ and the coefficient of the interaction were below 0.05, indicating a significant interaction effect between the two. It should be noted that the main effect coefficient between green travel behavior and trans-education was more than 0, while the coefficient of the interaction term was <0. This implies that too much self-efficacy may inhibit the positive effect of trans-education on green travel behavior, resulting in an overall effect that is less pronounced than expected.

In summary, trans-education can have a profound change in the green travel behavior of residents, not only by empowering them to opt for environmentally friendly and low-carbon travel modes but also by fostering a deeper commitment to environmental protection that reinforces sustainable behavioral patterns. Precisely, this form of education can positively influence residents' emotional disposition, normative beliefs, and personal continued green and low-carbon travel practices. Notably, our study, in contrast to previous research, reveals an intriguing finding, high self-efficacy, paradoxically, dampens the effectiveness of trans-education. This observation paves the way for future researches to delve deeper into this phenomenon and explore potential explanations.

### 6.1 Policy recommendations

The family is the most basic unit in society, and this study verifies the possibility of exploring the possibility of guiding residents to travel green from a family perspective. Based on the findings of the study, the following recommendations are made.

(1) Deliver richer green information to the younger generation through various channels, including school education, to cultivate a deep green mindset. Schools should strengthen collaborations with local environmental departments and organizations, inviting environmental experts to conduct lectures and provide more comprehensive green information. We should be good at stimulating students' sense of self-radiation and enriching the subject education on ecological construction and environmental protection actions when conducting instructional design. It is also important to design instructional designs that encourage students to motivate their parents. Subsequently, with the power of trans-education, the younger generation can become an important medium to push their parents to change their green travel behavior.

(2) The power of commitment should not be underestimated. The commitment made by the younger generation will have a far-reaching impact on their green and low-carbon behaviors. Similarly, the older generation's commitment to environmental protection will also positively impact their green travel behavior. Government agencies should organize more family-based environmental seminars and sharing sessions, encouraging the older generation to make environmental commitments during these events. This approach can enhance their environmental awareness and motivate them to serve as role models for younger generations in adopting green and low-carbon lifestyles.

(3) To cultivate a green and low-carbon culture in society, we need to recognize that overconfidence may lead to self-justifying motives, thus hindering the transformation of green travel behaviors. To overcome this obstacle, we must integrate green and low-carbon behaviors into societal norms and ethics, reinforcing residents' sense of responsibility. By enacting and enforcing relevant laws and policies, strengthening environmental education and advocacy, and establishing incentives for green travel, we can enhance citizens' environmental accountability and moral awareness. Furthermore, fostering collaboration among governments, businesses, and the public to build a culture of green and low-carbon living will create a favorable atmosphere where residents naturally opt for environmentally friendly modes of transportation. In this context, residents will more consciously choose green travel options, contributing to the sustainable development of society.

## Data Availability

The raw data supporting the conclusions of this article will be made available by the authors, without undue reservation.
